# SHP-1 is a target of regorafenib in colorectal cancer

**DOI:** 10.18632/oncotarget.2191

**Published:** 2014-07-09

**Authors:** Li-Ching Fan, Hao-Wei Teng, Chung-Wai Shiau, Hang Lin, Man-Hsin Hung, Yen-Lin Chen, Jui-Wen Huang, Wei-Tien Tai, Hui-Chuan Yu, Kuen-Feng Chen

**Affiliations:** ^1^ Department of Medical Research, National Taiwan University Hospital, Taipei, Taiwan; ^2^ National Center of Excellence for Clinical Trial and Research, National Taiwan University Hospital, Taipei, Taiwan; ^3^ Division of Hematology and Oncology, Department of Medicine, Taipei Veterans General Hospital, Taipei, Taiwan; ^4^ National Yang-Ming University School of Medicine, Taipei, Taiwan; ^5^ Institute of Biopharmaceutical Sciences, National Yang-Ming University, Taipei, Taiwan; ^6^ Program in Molecular Medicine, School of Life Sciences, National Yang-Ming University, Taipei, Taiwan; ^7^ Department of Pathology, Cardinal Tien Hospital, School of Medicine, Fu-Jen Catholic University, New Taipei, Taiwan; ^8^ Industrial Technology Research Institute, Hsin-Chu, Taiwan

**Keywords:** SHP-1, Regorafenib, Colorectal cancer, STAT3, Apoptosis

## Abstract

Regorafenib is an inhibitor of multiple protein kinases which exerts antitumor and antimetastatic activities in metastatic colorectal cancer (CRC). SH2 domain-containing phosphatase 1 (SHP-1) is reported to have tumor suppressive potential because it acts as a negative regulator of p-STAT3^Tyr705^ signaling. However, little is known about the mechanism regarding regorafenib affects SHP-1 tyrosine phosphatase activity and leads to apoptosis and tumor suppression in CRC. Here, we found that regorafenib triggered apoptotic cell death and significantly enhanced SHP-1 activity, which dramatically decreased the phosphorylated form of STAT3 at Tyr705 (p-STAT3^Tyr705^). Importantly, regorafenib augmented SHP-1 activity by direct disruption of the association between N-SH2 and catalytic PTP domain of SHP-1. Deletion of the N-SH2 domain (dN1) or point mutation (D61A) of SHP-1 blocked the effect of regorafenib-induced SHP-1 activity, growth inhibition and a decrease of p-STAT3^Tyr705^ expression, suggesting that regorafenib triggers a conformational change in SHP-1 by relieving its autoinhibition. *In vivo* assay showed that regorafenib significantly inhibited xenograft growth and decreased p-STAT3^Tyr705^ expression but induced higher SHP-1 activity. Collectively, regorafenib is a novel SHP-1 agonist exerts superior anti-tumor effects by enhancing SHP-1 activity that directly targets p-STAT3^Tyr705^. Small molecule-enhancement of SHP-1 activity may be a promising therapeutic approach for CRC treatment.

## INTRODUCTION

Colorectal cancer (CRC) is a leading malignancy worldwide. In general, the incidence of CRC is higher in economically developed countries than in developing countries [1]. Early stage CRC is usually treated with surgery, frequently in combination with adjuvant chemotherapy. Regorafenib is a novel oral multikinase inhibitor of both intracellular and membrane-bound receptor-tyrosine kinases (RTKs), which involved in the signal pathways related to tumor angiogenesis, oncogenesis, tumor microenvironment and tumor growth/proliferation [2, 3]. In preclinical studies, regorafenib exhibited antitumor activity in multiple tumor xenografts [2]. Recently, regorafenib demonstrated overall survival benefits in metastatic CRC patients and subsequently became the first approved therapeutic drug for the disease [3]. Regorafenib has been reported to not only exert antitumor effects by inhibiting cell proliferation and markedly suppressing xenograft growth, but also display antimetastatic and antiangiogenic activities in CRC [4].

Regorafenib (Fluoro-sorafenib) is structurally very similar to sorafenib, but regorafenib contains a fluorine atom rather than a hydrogen atom on the central aromatic ring. Sorafenib is also a multi-kinase growth inhibitor and is the first FDA-approved oral therapy for hepatocellular carcinoma (HCC) [5]. Recently, it has been demonstrated that sorafenib and its derivatives directly enhance the activity of Src homology region 2 (SH2) domain-containing phosphatase 1 (SHP-1) and which specifically decreases the phosphorylated form of STAT3 at Tyr705 (p-STAT3^Tyr705^) and eventually leads to cancer apoptosis and suppressed tumorigenicity [6, 7]. SHP-1 is first identified in hematopoietic cells and is predominantly expressed in hematopoietic and epithelial cells [8-10]. SHP-1 belongs to a family of non-receptor protein tyrosine phosphatases (PTPs), which involved in hematopoietic signaling processes and has also been reported to function as a tumor suppressor during tumor progression [11-14]. SHP-1 contains two SH2 domains, a catalytic PTP domain and a C-terminal tail [11]. Notably, its phosphatase activity is highly dependent on its structural variability. For example, the closed-form chemical structure of SHP-1 is assembled by the N-SH2 domain and protrudes into the catalytic domain to directly block the entrance into the active site, and the highly mobile C-SH2 domain is believed to act as an antenna to search for the phosphopeptide activator [15-19]. Other reports also indicate that the PTP catalytic domain is activated due to the flexibility of the WPD loop, which contains the active-site residue, Asp421 [11, 15, 16, 20].

Our recent work has mechanistically demonstrated that sorafenib-enhanced SHP-1 activity is due to the docking potential of sorafenib, which directly disrupts the association between N-SH2 and catalytic PTP domain of SHP-1 and further relieves SHP-1's autoinhibition [6, 7]. Importantly, our previous studies have clearly demonstrated that SHP-1 is a negative regulator of p-STAT3^Tyr705^ [6, 21-24] that is highly expressed in cancer cells and is crucial for growth, survival, and metastasis of cancer cells during cancer progression. Therefore, we clarify whether the sorafenib analog regorafenib (Fluoro-sorafenib) acts as a direct enhancer of SHP-1 by which enhanced SHP-1 activity directly downregulates p-STAT3^Tyr705^ and eventually contributes to apoptosis in CRC. Understanding the mechanism of the tumor suppression pathway activated by regorafenib may provide a new therapeutic strategy through which to abolish STAT3 oncogenic signaling. This study is aim to gain further insight into the molecular mechanisms through which regorafenib induces apoptosis *in vitro* as well suppresses tumorigenicity *in vivo*. To our knowledge, this is the first study to report that regorafenib is a novel SHP-1 agonist that directly impairs the interaction between the N-SH2 domain and the PTP catalytic domain of SHP-1, and further relieves the autoinhibition of SHP-1. Our study reveals the novel molecular mechanism through which regorafenib triggers an SHP-1 tumor suppressive pathway to exert superior anti-tumor effects by promoting SHP-1 tyrosine phosphatase activity that targets the oncogenic expression of p-STAT3 ^Tyr 705^ directly.

## RESULTS

### Regorafenib showed superior growth inhibition and significantly induced apoptosis in colon cancer cell lines

**Figure 1:**
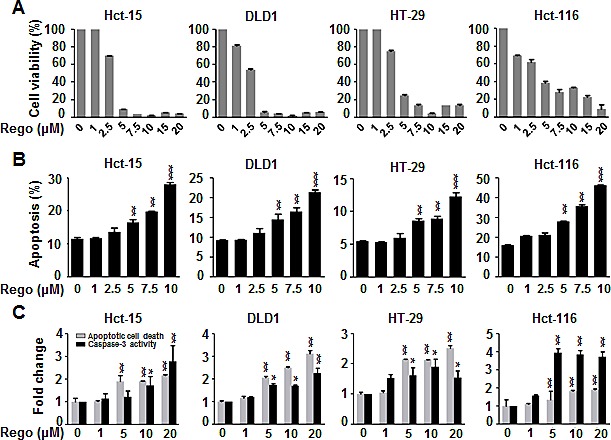
Regorafenib dramatically decreased cell viability and significantly induced apoptosis in colon cancer cell lines (A) MTT assay was performed to measure the cell viability in the colon cancer cell lines 2 days after treatment with regorafenib in a dose-dependent manner. (B) Sub-G1 analysis was used to detect the apoptosis in the cells 2 days after treatment with regorafenib in a dose-dependent manner. (C) 24-hours after cells were treated with regorafenib, apoptotic cell death and caspase-3 activity were significantly induced in a dose-dependent manner. Columns, mean; bars, SD. *P < 0.05, **P < 0.01, ***P < 0.001.

First, we investigated the biological effect of regorafenib on growth inhibition in a panel of 4 human CRC cell lines (Hct-15, DLD1, HT-29 and Hct-116). Two days after treatment with regorafenib at doses of more than 5 μM, cell viability was dramatically decreased (Figure 1A) and apoptosis was significantly induced (Figure 1B) in these CRC cell lines. Moreover, 24-hours after cells were treated with regorafenib, marked apoptotic cell death and increased specific caspase-3 activity occurred in a dose-dependent manner (Figure 1C). These findings suggest that regorafenib exerts an anti-cancer effect by inducing apoptotic cell death in CRC cells.

### Downregulated expression of p-STAT3^Tyr705^ was associated with the apoptotic effect of regorafenib

**Figure 2:**
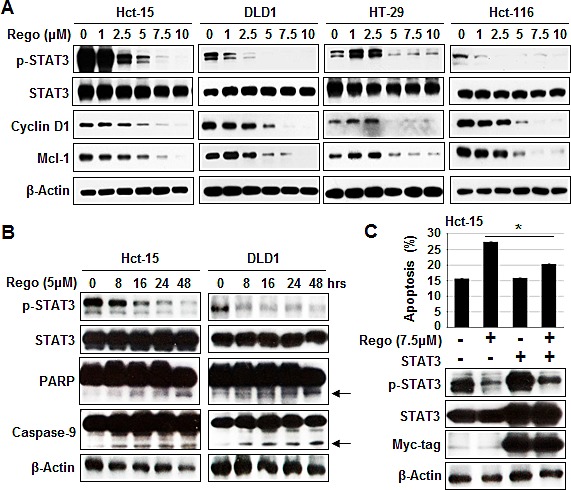
Sensitizing effects of regorafenib were associated with p-STAT3 (Tyr 705) downregulation (A) Western blotting of p-STAT3^Tyr705^ and its related survival markers cyclin D1 and Mcl-1 in cells 24-hours after treatment with regorafenib. β-actin was used as a loading control. (B) p-STAT3^Tyr705^, STAT3, the cleaved fragments of PARP and the cleaved fragments of caspase-9 were measured by western blotting at the times indicated after Hct-15 and DLD1 cells were treated with regorafenib at 5 μM. β-actin was used as a loading control. The cleaved fragments of PARP and the cleaved fragments of caspase-9 were indicated by arrows.(C) Overexpression of STAT3 rescued apoptosis induced by treatment with regorafenib at 5 μM for 24 hours. Columns, mean; bars, SD. *P < 0.05.

The phosphorylated form of STAT3 at Tyr^705^ (p-STAT3^Tyr705^) has been reported to be constitutively expressed in CRC and be involved in proliferative regulation [25]. Therefore, we next examined whether p-STAT3^Tyr705^ expression is involved in the apoptosis induced by regorafenib. Treatment of CRC cells with regorafenib for 24 hours gradually decreased the expression of p-STAT3^Tyr705^ and its related downstream survival targets cyclin D1 and Mcl-1 in a dose-dependent manner (Figure 2A). We also found that Hct-15 and DLD1 cells treated with regorafenib at 5μM gradually suppressed activated expression of p-STAT3^Tyr705^ but markedly increased apoptotic indicators the expressions of cleaved PARP and cleaved caspase-9 in a time-dependent manner (Figure 2B). Notably, overexpression of STAT3 obviously restored p-STAT3^Tyr705^ expression and inhibited apoptosis induced by treatment with regorafenib for 24 hours (Figure 2C), suggesting that regorafenib-triggered apoptosis is mediated by STAT3 inactivation.

### Regorafenib increased SHP-1 activity directly

**Figure 3:**
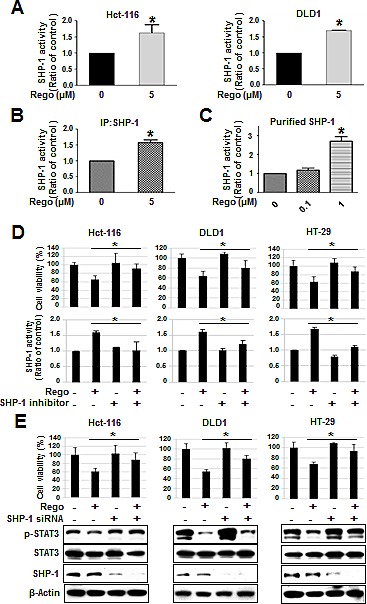
Regorafenib-enhanced SHP-1 tyrosine phosphatase activity was crucial for growth inhibition induced by regorafenib (A) SHP-1 activity was measured in the cells 24-hours after treatment with or without regorafenib. (B) Regorafenib increased the SHP-1 activity in IP-SHP-1-containing cell extract. (C) Regorafenib directly increased the SHP-1 activity in purified SHP-1 recombinant protein. Regorafenib-induced growth inhibition was depended on SHP-1 assessed by using 20 nM of SHP-1 phosphatase-specific inhibitor (PTPIII) (D) and 25 nM of siRNA specifically depleted SHP-1 (E). Columns, mean; bars, SD. *P < 0.05.

Ample evidence has demonstrated that the tyrosine phosphatase activity of SHP-1 significantly downregulates p-STAT3^Tyr705^ expression and induces apoptosis in different cancer types [6, 21-25]. Therefore, we evaluated whether SHP-1 activity is crucial for the dephosphorylation of STAT3 at Tyr^705^ induced by regorafenib. We found that regorafenib significantly increased SHP-1 tyrosine phosphatase activity in Hct-116 and DLD1 cells (Figure 3A). In addition, regorafenib-enhanced SHP-1 activity was observed in SHP-1-containing IP extract at 5 μM (Figure 3B). Notably, we further verified regorafenib augmented SHP-1 activity by incubation with cell-free purified SHP-1 protein (Figure 3C). Taken together, these results suggest that regorafenib is a novel SHP-1 agonist that increases SHP-1 activity in CRC. In order to know whether the regorafenib-induced growth inhibition is depended on SHP-1, we hence used SHP-1 phosphatase-specific inhibitor (PTPIII) to inhibit SHP-1 activity and found that regorafenib-induced growth inhibition in CRC cell lines were significantly rescued by treatment with SHP-1 inhibitor (Figure 3D). In addition, siRNA-mediated SHP-1 depletion in CRC cell lines also significantly reduced the effects of regorafenib on growth inhibition and p-STAT3^Tyr705^ expression (Figure 3E). These results suggest that SHP-1 was crucial for regorafenib-induced growth inhibition in CRC cells.

### Regorafenib enhanced SHP-1 tyrosine phosphatase activity by directly impairing the interaction between the N-SH2 and PTP catalytic domains of SHP-1

**Figure 4:**
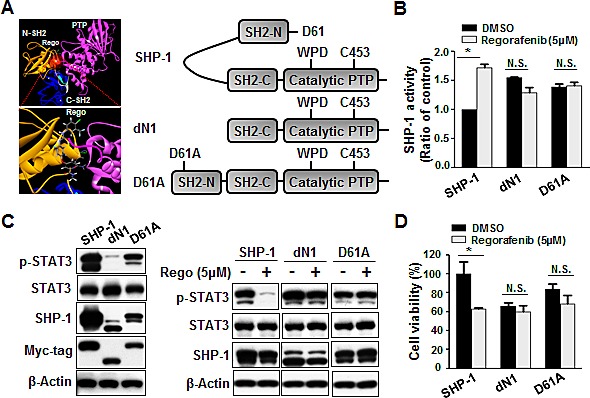
Regorafenib potently relieved the autoinhibition of SHP-1 and contributed to growth inhibition (A) Left: Modeled docking of regorafenib into the N-SH2 site of SHP-1 (pdb code: 3PS5). The N-SH2 domain was in gold, the C-SH2 domain was in marine, the PTP domain was in hot pink, and the linkers between them were in gray. The small-molecule docking site (by CDOCKER), which was labeled by a transparent red circle, was around the N-SH2 domain and C-terminal residues. Regorafenib formed a hydrogen bond (shown in green dashed lines) with Gln527. The -CDOCKER interaction energy (CDOCKER docking score) was 40.38. Right: A schematic representation of wild-type and mutant SHP-1 (D61A, a single point mutation, and dN1 with a deletion of the N-terminal inhibitory domain). (B) Two days after cells were treated with regorafenib at 5 μM, SHP-1 activity was potently increased in wild-type SHP-1-expressing but not in dN1 or D61A mutant SHP-1-expressing cells. *Columns*, mean; bars, SD. *P < 0.05 (C). Left: dN1 and D61A mutant SHP-1 with relatively higher SHP-1 activity showed relatively lower expression of p-STAT3^Tyr705^ compared with wild-type SHP-1. Right: dN1 and D61A mutant SHP-1 blocked the effects of regorafenib on reduced p-STAT3^Tyr705^ expression and growth inhibition. (D). MTT assay was performed to measure the cell viability. Columns, mean; bars, SD. *P < 0.05

Next, in order to understand the mechanism through which regorafenib regulates SHP-1 tyrosine phosphatase activity, we provide a molecular rationale for the docking model that regorafenib docks into the interface of N-SH2 and PTP domain which is crucial for SHP-1 activation. The small-molecule docking site (by CDOCKER), which is labeled by a transparent red circle, is around the N-SH2 domain and C-terminal residues. Regorafenib forms a hydrogen bond (shown in green dashed lines) with Gln527. The -CDOCKER interaction energy (CDOCKER docking score) is 40.38. (Figure 4A, left). Therefore, we subsequently transfected Hct-116 cells with wild-type or SHP-1 mutants dN1 and D61A, and further analyzed SHP-1 activity as well as the susceptibility to STAT3 dephosphorylation at Tyr705. We focused particularly on the intramolecular inhibition (salt bridge) between Asp61 (D61) within the N-SH2 domain, and Lys362 contained in the PTP catalytic domain of SHP-1 (Figure 4A, right). The D61A mutant mimics the open conformation of SHP-1 and dN1 is the deletion of N-SH2 domain of SHP-1, and both serve as constitutive activators.

Indeed, dN1 and D61A mutant SHP-1-overexpressing cells showed the relatively higher SHP-1 activity (Figure 4B) but displayed the relatively lower viability (Figure 4D) and a decrease of p-STAT3 expression, compared with wild-type SHP-1-expressed cells (Figure 4C, left). Notably, regorafenib significantly enhanced SHP-1 activity in wild-type SHP-1-transfected cells, but not in D61A or dN1 mutant SHP-1-transfected cells (Figure 4B), suggesting that regorafenib increases SHP-1 activity by direct disruption of the association between the N-SH2 domain and the PTP catalytic domain. We also observed drastically downregulated p-STAT3^Tyr705^ expression and significant inhibition of growth in regorafenib-treated wild-type SHP-1 expressing cells but no significant p-STAT3^Tyr705^ expression either in D61A or dN1 mutant SHP-1-transfected cells following treatment with regorafenib (Figure 4C, right and D). Together these results demonstrate that regorafenib affects SHP-1 activity by its potential to dock to the inhibitory N-SH2 domain and the catalytic PTP domain of SHP-1. Regorafenib, therefore, appears to be a novel SHP-1 agonist that not only directly relieves the autoinhibition of SHP-1 but further specifically increases susceptibility to STAT3 dephosphorylation at Tyr705 by regorafenib-enhanced SHP-1 activity.

### The suppressive effect of regorafenib on tumor formation *in vivo*

**Figure 5:**
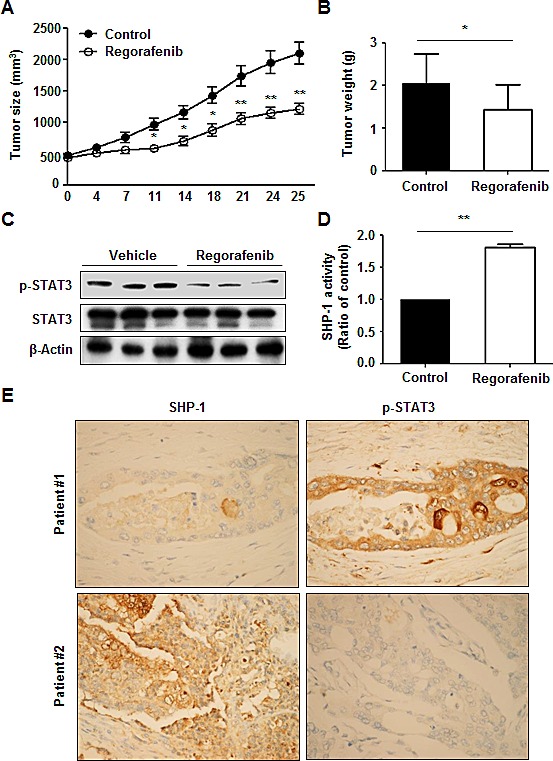
Regorafenib had a suppressive effect on tumor formation *in vivo* (A) Tumor sizes were measured at the times indicated after treatment with or without regorafenib at 10mg/kg/day. *Points*, mean (n = 10); *bars*, SE. *P < 0.05, **P < 0.01. (B) Tumor weight was measured on day 25 after tumor excision. *Columns*, mean; bars, SD. *P < 0.05 (C) The levels of p-STAT3^Tyr705^ and STAT3 were measured by western blotting on day 25 after excision of vehicle- and regorafenib-treated tumors. β-actin was used as a loading control. (D) SHP-1 activity was measured in vehicle- and regorafenib-treated tumors on day 25 after tumor excision. Columns, mean; bars, SD. *P < 0.05. (E) SHP-1. and p-STAT3^Tyr705^ immunohistochemical staining in two clinical CRC samples.

**Figure 6:**
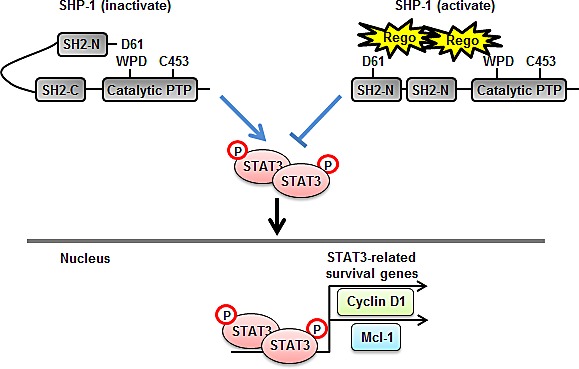
A representation of relief of the inhibitory N-SH2 domain of SHP-1 by regorafenib and its contribution to apoptosis in CRC Regorafenib enhanced SHP-1 tyrosine phosphatase activity by its potential to dock to the inhibitory N-SH2 domain and the catalytic PTP domain of SHP-1, thus, contributing to the direct relief of autoinhibition in SHP-1 but further specifically increased susceptibility to STAT3 dephosphorylation at Tyr705 thereby downregulating the transcriptional activation of survival genes, such as Cyclin D1 and Mcl-1, consequently, leading to the occurrence of apoptosis in CRC.

To investigate whether regorafenib has a therapeutic effect on tumorigenesis *in vivo*, we subcutaneously injected Hct-116 cells (2 × 10^6^) into the posterior flank of nude mice and then treated them with or without regorafenib at 10 mg/kg/day when the tumors became palpable and grew rapidly. The tumor size of regorafenib- and vehicle-treated tumors were measured on days 0, 4, 7, 11, 14, 18, 21, 24 and 25 and it was found that regorafenib-treated tumors grew slowly compared with vehicle-treated tumors (Figure 5A). On day 25 after treatment with regorafenib, primary tumors were excised and measured. Vehicle-treated tumors were heavier than regorafenib-treated tumors (Figure 5B), which suggests that regorafenib had a suppressive effect on tumor formation. Notably, regorafenib-treated tumors showed a relatively lower expression of p-STAT3^Tyr705^ (Figure 5C) but a significantly higher SHP-1 tyrosine phosphatase activity, compared with vehicle-treated tumors (Figure 5D), suggesting that suppression of tumorigenicity by regorafenib *in vivo* is due to its enhancement of SHP-1 activity that directly targeted p-STAT3^Tyr705^ expression. Immunohistochemistry staining of SHP-1 and p-STAT3^Tyr705^ in two CRC patients also showed that the clinical CRC patient#1 with strong positive expression of p-STAT3^Tyr705^ had weak positive expression of SHP-1. In contrast, patient#2 with weak positive expression of p-STAT3^Tyr705^ had strong positive expression of SHP-1 (Figure 5E). However, whether there is an inverse correlation between SHP-1 and p-STAT3^Tyr705^ in CRC samples will need to perform the experiments on a larger number of patients. Therefore, further examination of the role of SHP-1 on a large number of CRC tissues is required. Collectively, our findings establish a novel role of regorafenib as an agonist of SHP-1 tyrosine phosphatase activity by direct disruption of the association between N-SH2 and catalytic PTP domain of SHP-1 and then downregulating the expressions of p-STAT3^Tyr705^ and its related survival factors such as Cyclin D1 and Mcl-1, thereby, triggering apoptotic induction in CRC (Figure 6).

## DISCUSSION

Regorafenib is a novel multikinase inhibitor that has recently become the first approved treatment for CRC [2, 3]. Here, we provided further insight into the molecular mechanisms through which regorafenib potently induces apoptosis in CRC cells *in vitro* and suppresses tumorigenicity *in vivo*. We also provided the first evidence demonstrating that regorafenib is a novel enhancer of SHP-1 tyrosine phosphatase activity, which dramatically decreases p-STAT3^Tyr705^ expression. Ample evidence suggests that SHP-1 functions as a tumor suppressor that directly targets the oncogenic expression of p-STAT3^Tyr705^ that is crucial for the tumor survival and cellular proliferation [6, 21-24]. Our study demonstrated for the first time that regorafenib activated an SHP-1 tumor suppression pathway by directly enhancing SHP-1 activity, and induced apoptosis and tumor suppression in CRC.

SHP-1 is predominantly expressed in hematopoietic and epithelial cells [8-10] and its phosphatase activity is highly dependent on its structural variability, as evidenced by its open- and closed-form chemical structure [15-19]. The crystal structure of ligand-free SHP-1 engages in auto-inhibition due to intramolecular interaction between its N-SH2 domain and catalytic PTP domain. Notably, the specific residue, D61, forms a critical salt bridge resulting in a “closed” catalytic PTP domain. Our study showed that regorafenib-enhanced SHP-1 activity was significantly observed in CRC cells and was also seen in SHP-1 containing IP extract at 5 μM. Cell-free purified SHP-1 protein further verified the significantly increased SHP-1 activity directly enhanced by regorafenib. Importantly, we also observed the significant induction of SHP-1 activity by overexpressing wild-type SHP-1 in CRC cells. This induction was not seen in D61A and dN1 mutant SHP-1-expressing CRC cells, indicating that regorafenib-enhanced SHP-1 activity is due to its docking potential between the inhibitory N-SH2 domain and catalytic PTP domain of SHP-1, which directly relieves the autoinhibition of SHP-1. We also observed regorafenib dramatically downregulated p-STAT3^Tyr705^ expression and significant suppression of growth in wild-type SHP-1-expressing CRC cells, but not in D61A and dN1 mutant SHP-1-expressing CRC cells, suggesting that the increased susceptibility to STAT3 dephosphorylation at Tyr705 is due to regorafenib-enhanced SHP-1 activity.

In this study, we validated the role of SHP-1 on the *in vitro* effect of regorafenib by using SHP-1 phosphatase-specific inhibitor and siRNA to inhibit SHP-1 activity and knockdown its expression, respectively. Both of which significantly reduced the effects of regorafenib on growth inhibition in CRC cells, indicating that SHP-1 not only as a crucial player of regorafenib-induced growth inhibition, but also as a direct target of regorafenib by which enhanced SHP-1 tyrosine phosphatase activity downregulated p-STAT3^Tyr705^ directly. Although we validated the role of SHP-1 in regorafenib-triggered apoptosis or growth inhibition *in vitro*, the sufficient data from *in vivo* animal study is still lack. To generate lentiviruses that produced specific short-hairpin RNA (shRNA) targeting SHP-1, which will be a good approach to further validate the role of SHP-1 on anti-tumor effect of regorafenib *in vivo*.

To our knowledge, this is the first study to reveal the role of regorafenib as a novel SHP-1 agonist in a preclinical model of CRC. We also discovered that SHP-1 tumor suppression pathway-mediated apoptosis and anti-tumor activity was significantly triggered by regorafenib. Our studies suggest that SHP-1 may be a useful biomarker for predicting the efficacy of regorafenib treatment in CRC patients.

## METHODS

### Cell culture

Colorectal cancer (CRC) cell lines Hct-15, DLD1, HT-29 and Hct-116 were maintained in RPMI 1640 medium supplemented with 10% fetal bovine serum (FBS) and 100 units/ml of penicillin and streptomycin (Invitrogen, Carlsbad, CA, USA). Cells were then incubated at 37°C in a humidified 5% CO_2_ atmosphere.

### Reagents, plasmid and test kit

Regorafenib was kindly provided by Bayer HealthCare Pharmaceuticals. For cell-based studies, regorafenib at various concentrations was dissolved in DMSO and then added to the cells maintained in RPMI 1640 medium with or without FBS. SHP-1 inhibitor (PTP III) was purchased from Calbiochem. Smart-pool siRNA, including control (D-001810-10), SHP-1 (PTPN6, L-009778-00-0005) were all purchased from Dharmacon Inc. (Chicago, IL). Plasmids of human wild-type STAT3 and SHP-1 (PTPN6) were encoded by pCMV6 vector with myc-tag. For mutant-type SHP-1 expression, we generated two plasmids, designated dN1 and D61A, which truncated the N-SH2/PTP domain and changed aspartic acid at 61 to an alanine residue, respectively. Both plasmids were cloned into pCMV6-Entry vector. These plasmids or siRNA were subsequently transfected into cells by using Lipofectamine 2000 reagent (Invitrogene, CA). Apoptotic cell death was determined using the Cell Death Detection ELISA^PLUS^ kit (Roche Applied Sciences, Germany). Caspase-3 activity was measured using the Caspase 3 Assay Kit (Abcam, Cambrige, MA). Both of the assays were carried out according to the manufacturer's instructions.

### Cell proliferation assay

After treatment with regorafenib at the indicated doses for 2 days, cell proliferation was measured using a 3-(4, 5-dimethylthiazol-2-yl)-2, 5-diphenyltetrazolium bromide (MTT) assay. Cells were counted and seeded in 96-well plates, and then incubated at 37°C in a humidified 5% CO_2_ atmosphere. Twenty microliters of MTT reagent (5 mg/ml, Sigma, St Louis, MO, USA) was added to each well at the end of incubation, then 4 hours later the medium was discarded and 150 μl dimethylsulfoxide (DMSO) was added to each well to dissolve the purple crystal. Then the absorbance at 490 nm was measured. Experiments were performed three times in duplicate.

### Apoptosis detection by sub-G1 analysis

For the analysis of apoptosis, both floating and attached cells were collected and centrifuged before being washed with cold phosphate-buffered saline (PBS), and then fixed in 70% cold ethanol overnight at -20°C. Propidium iodide staining was performed after incubation of the cells with 50 μg/ml propidium iodide and 20 μg/ml RNase A in the dark at room temperature for 30 min, which were then analyzed by flow cytometry (BD Biosciences, San Jose, CA, USA).

### Western blotting

Whole-cell lysates were resolved by sodium dodecyl sulfate polyacrylamide gel electrophoresis (SDS-PAGE). Proteins were transferred onto a polyvinylidene difluoride membrane (Millipore, Billerica, MA, USA) and incubated with the primary antibody, and then incubated with horseradish peroxidase-conjugated secondary antibodies. Specific proteins were detected using enhanced chemiluminescence (ECL) reagent. The primary antibodies used for western blotting, including cyclin D1 and PARP were purchased from Santa Cruz Biotechnology (San Diego, CA); p-STAT3, STAT3, Mcl-1 and Caspase-9 were purchased from Cell Signaling (Danvers, MA); SHP-1 and β-actin were purchased from Abcam (Cambrige, MA).

### SHP-1 phosphatase activity

After treatment with regorafenib, cell protein extracts were incubated with anti-SHP-1 antibody in immunoprecipitation buffer (20 mM Tris-HCl (pH 7.5), 150 mM NaCl, 1mM EDTA, 1% NP-40, and 1% sodium deoxycholate) overnight. Protein G-Sepharose 4 Fast flow (GE Healthcare Bio-Science, NJ) was added to each sample, followed by incubation for 3 h at 4°C with rotation. A RediPlate 96 EnzChekR Tyrosine Phosphatase Assay Kit (R-22067) was used for SHP-1 activity assay (Molecular Probes, Invitrogen, CA).

### Xenograft tumor growth

We subcutaneously injected Hct-116 cells (2 × 10^6^) into the posterior flank of nude mice and then treated them with or without regorafenib at 10 mg/kg/day when the tumors became palpable and grew rapidly. The tumor size of regorafenib- or vehicle-treated tumors was measured on days 0, 4, 7, 11, 14, 18, 21, 24 and 25. On day 25 the tumor weights were measured after tumor excision.

### Immunohistochemistry

Specimens fixed in 10% buffered formalin were embedded in paraffin and then sectioned. Immunohistochemical analysis of the paraffin sections was done using primary antibody for SHP-1 and p-STAT3; the sections were then incubated with anti-mouse/rabbit immunoglobulin G-horseradish peroxidase-conjugated secondary antibody. The signal was detected using a kit (Aminoethyl Carbazole Substrate Kit; Zymed Laboratories Inc., San Francisco, CA, USA). The sections were counterstained with hematoxylin.

## SUPPLEMENTARY MATERIAL


