# Supplementary material for: SHP-1 is a target of regorafenib in colorectal cancer
Source: Oncotarget. 2014 Jul 9;5(15):6243–51. doi: 10.18632/oncotarget.2191 (PMC4171626; doi:10.18632/oncotarget.2191)
Supplement: Supplementary file 1 [file oncotarget-05-6243-s001.pdf]

SHP-1 is a target of regorafenib in colorectal cancer

Supplemental Material

Modeled docking of regorafenib

The molecular modeling job was performed by Discovery Studio 3.5 (Accelrys, San Diego, CA, USA). The crystal structure of SHP-1 was downloaded from Protein Data Base (pdb cod:3PS5). The protein was prepared by the program “prepare protein” (in Macromolecules protocol), and the docking site was identified by “Define Site” program (in Receptor-Ligand Ineractions protocol). There were 16 sites found, and we selected the site around N-SH2 domain. The docking was applied CDOCKER program. The forcefield is CHARMm and the Grid Extension is 8.0 Å. The other parameters were defaulted. The docking was evaluated by –CDOCKER interaction energy. The definicaiton of –CDOCKER interaction energy is that the nonbonded interaction energy between the ligand and the protein. A higher value of –CDOCKER interaction energy indicates a more favorable binding.[a]

[a]CDOCKER program introduction, by Accelrys.

http://accelrys.co.kr/about/news_topic/120224_Session1_Binding_Site_Analysis_and_CDOCKER.pdf
